# Supplementary material for: Integrative Analysis of DNA Methylation and microRNA Reveals GNPDA1 and SLC25A16 Related to Biopsychosocial Factors Among Taiwanese Women with a Family History of Breast Cancer
Source: J Pers Med. 2025 Mar 30;15(4):134. doi: 10.3390/jpm15040134 (PMC12028518; doi:10.3390/jpm15040134)
Supplement: Supplementary file 1 [file jpm-15-00134-s001.zip › jpm-3469819-supplementary.pdf]

Table S1. **Top 10 significantly CpGs of DEMs**

No	CpG sites	*p-value*	Log2FC	FDR
1	cg23786701	5.98×10^{-14}	-4.637	5.18×10^{-8}
2	cg11799704	3.99×10^{-13}	4.515	1.73×10^{-07}
3	cg00267373	1.37×10^{-12}	4.314	3.77×10^{-07}
4	cg16924658	1.47×10^{-12}	4.356	3.77×10^{-07}
5	cg15340709	1.04×10^{-11}	4.156	1.81×10^{-06}
6	cg18075755	2.41×10^{-11}	-2.797	3.47×10^{-06}
7	cg08078832	3.79×10^{-11}	4.096	4.69×10^{-06}
8	cg13120424	4.69×10^{-11}	4.066	4.84×10^{-06}
9	cg05951425	5.03×10^{-11}	-3.914	4.84×10^{-06}
10	cg14041264	1.25×10^{-10}	-3.825	1.08×10^{-05}

Notes: Log2FC (Log2 fold change); FDR (false discovery rate)

Table S2. **Top 10 significantly hsa-mir of DEmiRNA**

No	miRNA	*p-value*	Log2FC	FDR
1	hsa-let-7c-5p	4.15×10^{-46}	-1.518	4.22×10^{-43}
2	hsa-miR-664b-5p	2.77×10^{-41}	-2.962	1.41×10^{-38}
3	hsa-let-7b-5p	3.94×10^{-40}	-2.208	1.33×10^{-37}
4	hsa-miR-425-5p	3.79×10^{-38}	2.113	9.63×10^{-36}
5	hsa-miR-23a-3p	5.88×10^{-37}	1.373	1.19×10^{-34}
6	hsa-miR-4446-3p	4.42×10^{-34}	-4.039	7.49×10^{-32}
7	hsa-let-7d-3p	3.10×10^{-32}	-2.684	4.49×10^{-30}
8	hsa-miR-30e-3p	9.90×10^{-32}	1.908	1.26×10^{-29}
9	hsa-miR-21-5p	1.65×10^{-27}	2.273	1.87×10^{-25}
10	hsa-miR-3960	1.99×10^{-26}	-4.623	2.03×10^{-24}

Notes: Log2FC (Log2 fold change); FDR (false discovery rate)

Table S3. **Prognostic Value of Single CpG of the potential genes in breast cancer by MethSurv platform.** The threshold of significance was LR Test p-value <0.05. A significant expression pattern was found in 3 of 19 genes-CpGs between low and high-risk groups for breast cancer

Gene-CpG	HR	LR Test p-value
GNPDA1-TSS1500-S_Shore-cg00145118	0.506	0.0016
GNPDA1-TSS1500-S_Shore-cg05560697	0.848	0.41
GNPDA1-TSS200-S_Shore-cg05515516	1.146	0.55
GNPDA1-TSS200-S_Shore-cg07279858	0.88	0.52
GNPDA1-TSS200-S_Shore-cg10927534	1.164	0.52
GNPDA1-TSS200-S_Shore-cg15201400	0.705	0.18
GNPDA1-TSS200-S_Shore-cg22463008	1.222	0.37
GNPDA1-Body-N_Shelf-cg01609099	0.72	0.17
GNPDA1-5′UTR-Island-cg07296147	0.817	0.31
GNPDA1-5′UTR-Island-cg19704039	1.533	0.08
GNPDA1-3′UTR-Open_Sea-cg12374003	1.273	0.3
GNPDA1-Body-N_Shore-cg25940447	0.791	0.25
SLC25A16-5′UTR;1stExon-Island-cg00203461	2.109	0.0013
SLC25A16-5′UTR;1stExon-Island-cg12119218	1.478	0.094
SLC25A16-Body-Island-cg10590909	1.768	0.01
SLC25A16-1stExon-Island-cg17823321	0.774	0.28
SLC25A16-Body-N_Shore-cg01284033	0.74	0.13
SLC25A16-5′UTR;1stExon-S_Shore-cg14191109	0.69	0.12
SLC25A16-Body-Open_Sea-cg16214034	0.396	3.4E-05

Notes: HR (hazard ratio); LR (Likelihood Ratio); CI (confidence interval)

Table S4. **Breast Cancer Survival. The cohort of the TCGA database**

Variables	Patient number (N)	Univariate			Patient number (N)	Multivariate		
		HR	(95% CI)	p-value		HR	(95% CI)	p-value
Age								
< 60	552	Ref			552	Ref		
≥ 60	442	1.988	1.412-2.798	**<0.001**	442	1.774	1.110-2.835	**0.016**
Tumor stage								
Stage I	166	Ref			166	Ref		
Stage II	574	1.376	0.790-2.398	0.260	574	1.860	0.610-5.667	0.275
Stage III	218	2.840	1.588-5.078	**<0.001**	218	3.462	1.085-11.045	**0.036**
Stage IV	18	12.995	6.320-26.717	**<0.001**	18	6.225	1.527-25.378	**0.011**
Stage X	13	3.236	1.254-8.354	**0.015**	13	7.200	2.176-23.821	**0.001**
T_Stage								
T1	226	Ref			226	Ref		
T2	586	1.248	0.812-1.919	0.313	586	0.773	0.333-1.792	0.548
T3	113	1.624	0.928-2.842	0.089	113	0.604	0.224-1.634	0.321
T4	37	4.238	2.226-8.066	**<0.001**	37	1.185	0.370-3.801	0.775
Tx	3	0.625	0.084-4.658	0.647	3	0.213	0.021-2.145	0.189
Treatment								
No	719	Ref						
Yes	86	7.188	4.593-11.250	**<0.001**				

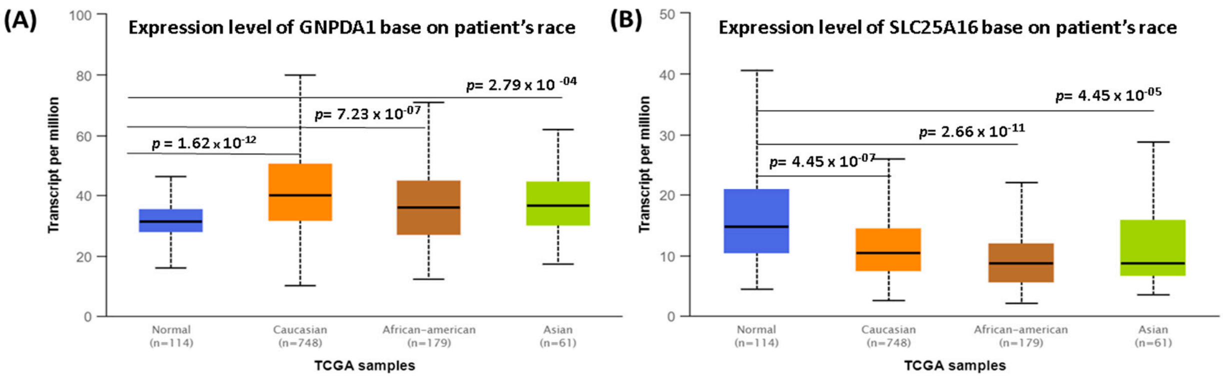

Figure S1. Expression level analysis of the potential genes based on the patient's race in breast cancer (TCGA database). (A-B) Box plot of the transcript levels of GNPDA1 and SLC25A16 in breast cancer patients from different ethnicities (Caucasian, African-American, and Asian), as compared with healthy women. Statistical significance was indicated by $p < 0.0001$

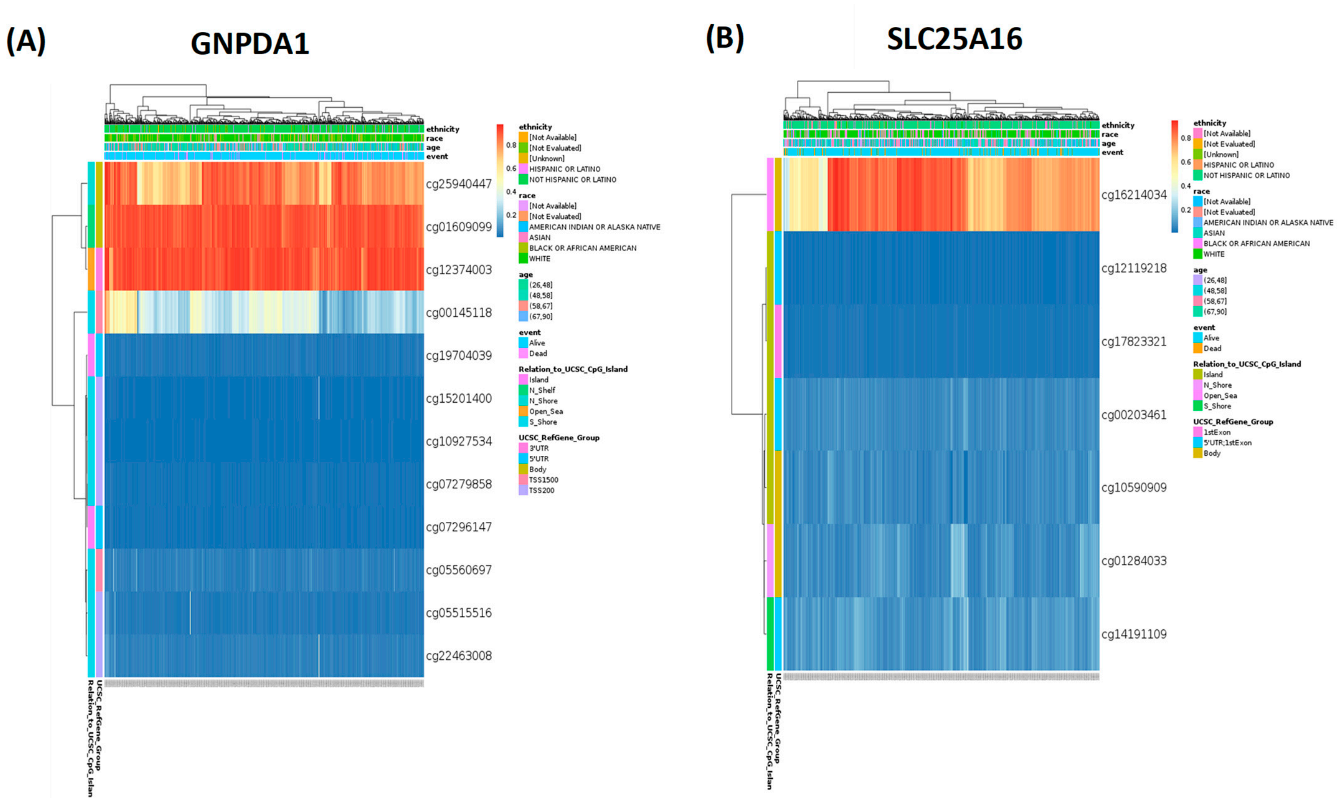

Figure S2. Heatmap of DNA methylation expression levels of the potential genes in breast cancer by MethSurv platform. cg25940447; cg01609099; cg12374003; cg00145118; cg19704039; cg15201400; cg10927534; cg07279858; cg07296147; cg05560679; cg05515516; and cg22463008 of GNPDA1, cg16214034; cg12119218; cg17823321; cg00203461; cg10590909; cg01284033; and cg14191109 of SLC25A16 display the level of DNA Methylation (hyper-methylation or hypo-methylation) in breast cancer.

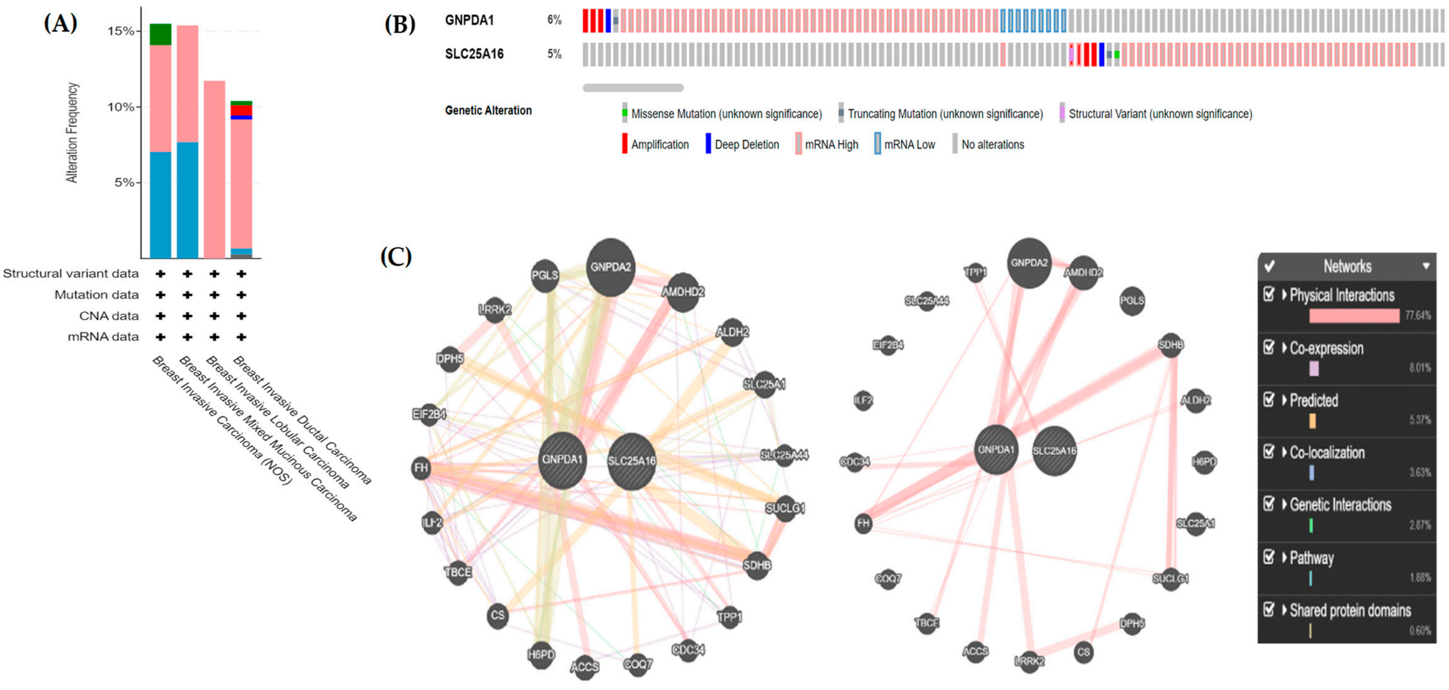

Figure S3. **Genetic alteration, neighbor gene network, and interaction analysis of the two potential genes in patients with breast cancer (cBioPortal and GeneMANIA)**. (A) Summary of alterations in various invasive breast carcinomas. (B) OncoPrint visual summary of alteration on two potential biomarkers. (C) Gene–gene interaction of GNPDA1 and SLC25A16 in the GeneMANIA data set.

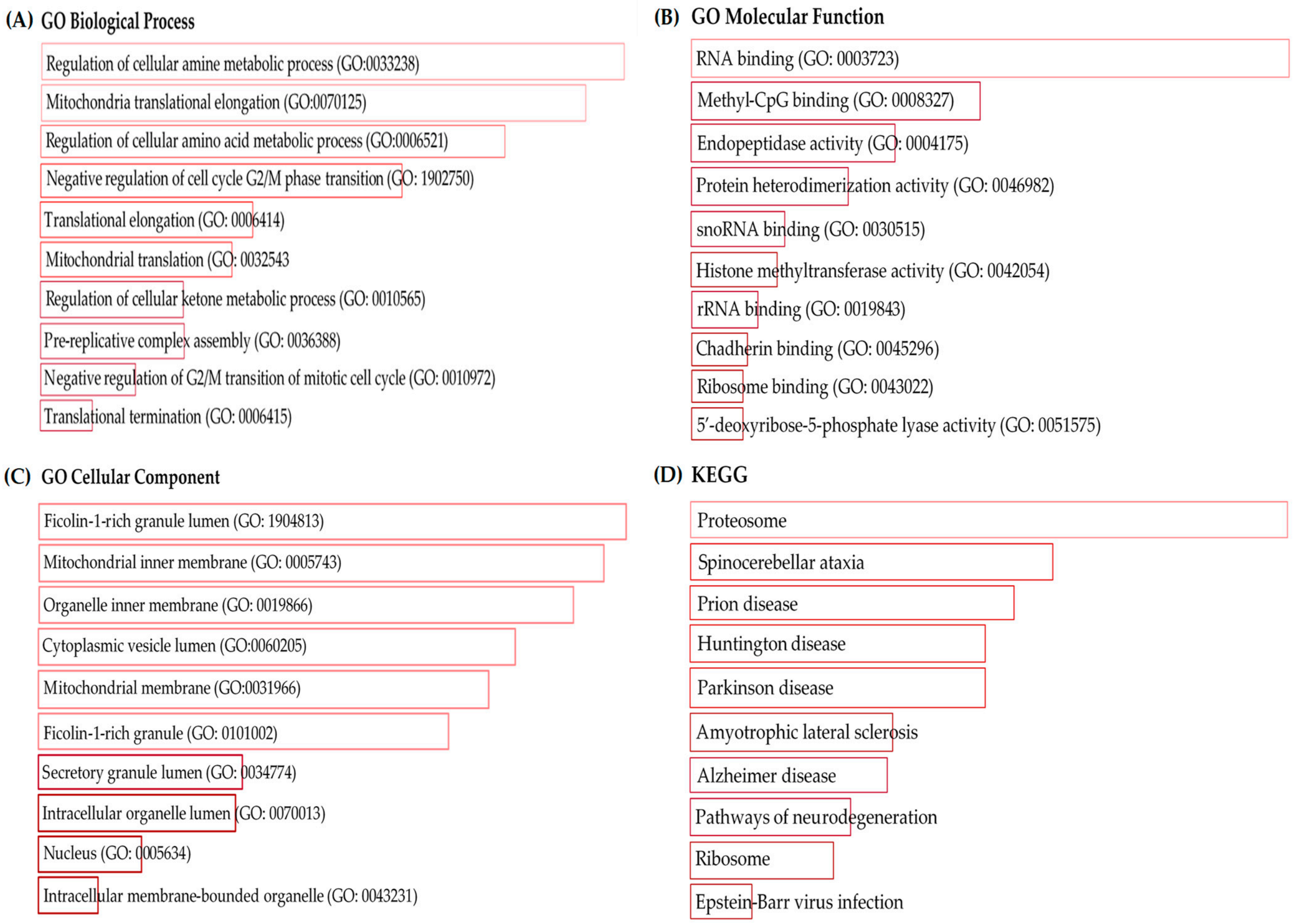

Figure S4. Gene ontology analysis based on genes co-expressed with GNPDA1 from the Enrichr database. (A) Biological processes. (B) Cellular components. (C) Molecular functions. (D) KEGG. ARCHS4 RNA-seq gene-gene co-expression matrix found the top 200 genes co-expressed with GNPDA1

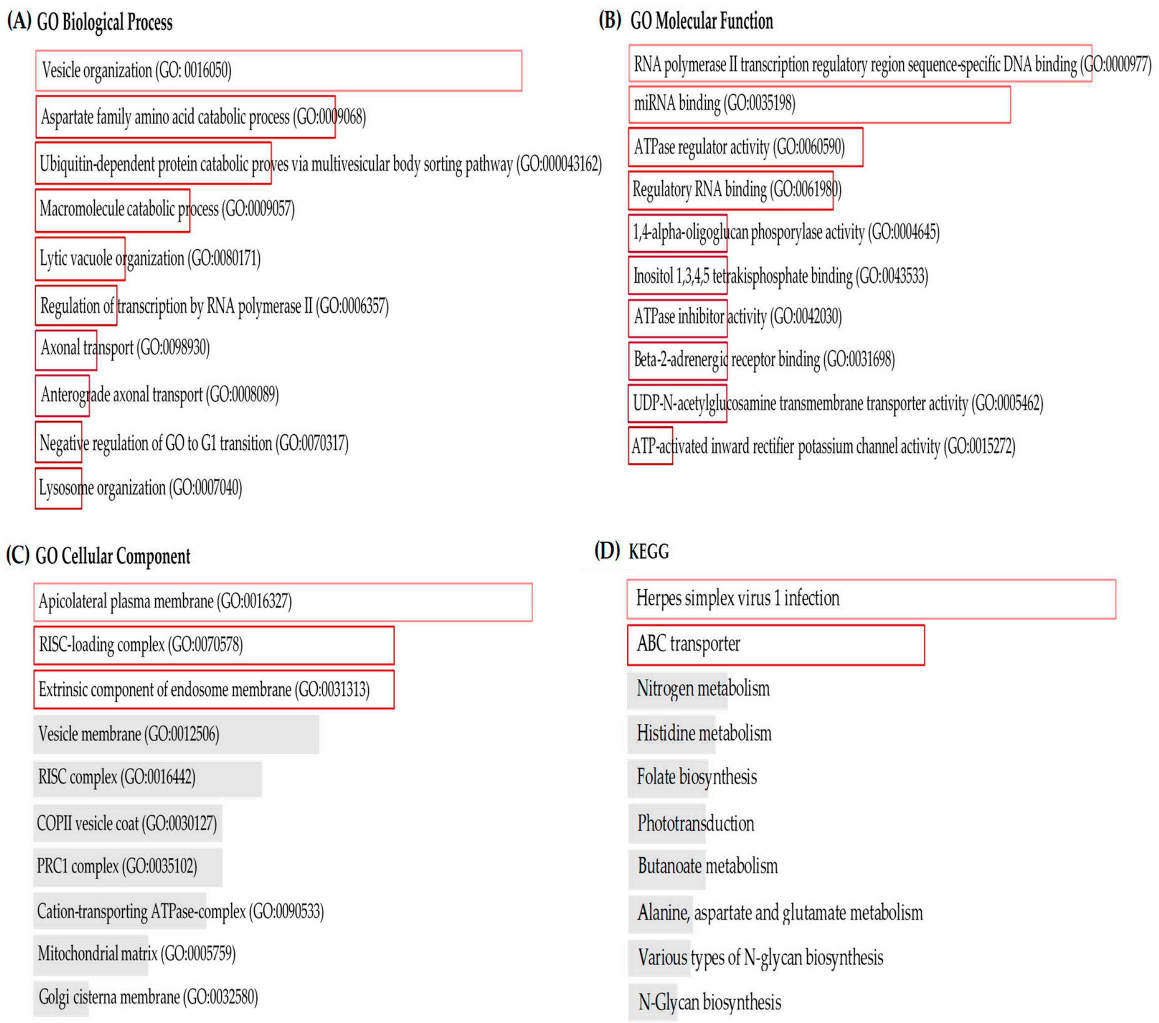

Figure S5. Gene ontology (GO) analysis based on genes co-expressed with *SLC25A16* from the Enrichr database. (A) Bar graph of biological processes. (B) Bar graph of cellular components. (C) Bar graph of molecular functions. (D) Bar graph of KEGG. The bar graph is determined by the Fisher exact test which is a proportion test that assumes a binomial distribution and independence for the probability of any gene belonging to any set.

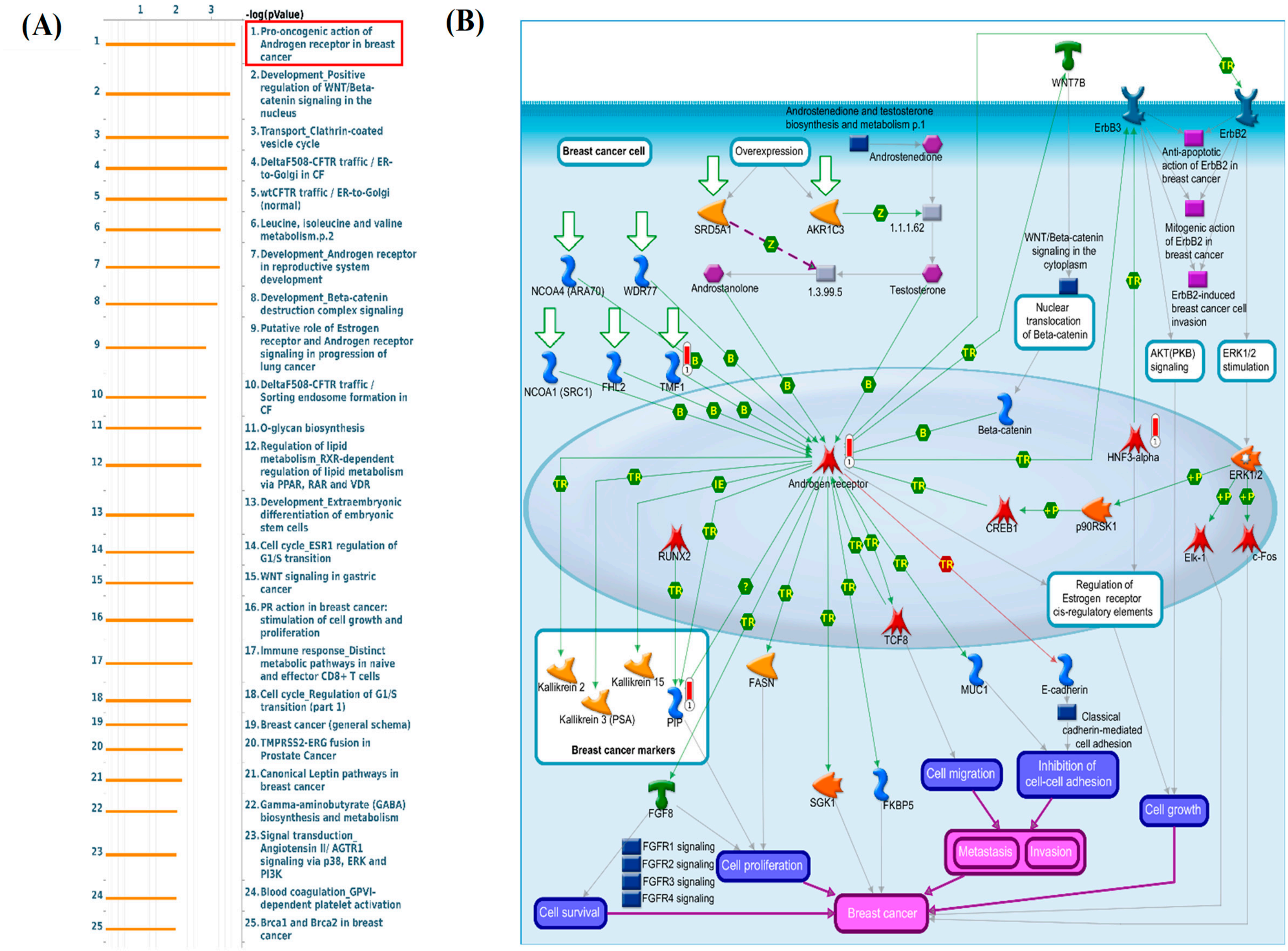

Figure S6. Expression of the SLC25A16 signaling pathway in breast cancer (MetaCore). We used the MetaCore platform to analyzed 10% genes co-expressed which have correlation value (Spearmen's partial rho = ≥ 0.3) with SLC25A16. (A) Bar graph of pathway distribution. We found that "Pro-oncogenic action of androgen receptor" were correlated with breast cancer development (with $p < 0.05$ set as the cutoff value). (B) Signaling pathway of Pro-oncogenic action of androgen receptor in breast cancer patients.

Table S5. **GNPDA1 differentially expressed genes pathway developed by MetaCore.** Brca1 and Brca2 was correlated with breast cancer development

#	Maps	pValue	Network Objects from Active Data
1	DNA damage_ATM activation by DNA damage	4.282E-06	OBFC2B, RAD17, INTS3, Brca1, PP2A catalytic, HP1 beta, Rad50
2	Brca1 and Brca2 in breast cancer	6.950E-04	Tubulin gamma, Brca1, Rad50
3	DNA damage_ATM/ATR regulation of G2/M checkpoint: nuclear signaling	8.093E-04	CDC25C, Brca1, Cyclin B, PALB2
4	Aminoacyl-tRNA biosynthesis in mitochondrion	9.611E-04	DARS2, SYHH, IARS2, TARSL1, EARS2
5	DNA damage_ATM/ATR regulation of G2/M checkpoint: cytoplasmic signaling	1.300E-03	CDC25C, Brca1, PP2A catalytic, PARN
6	Cell cycle_Initiation of mitosis	1.778E-03	CDC25C, FOXM1, CDK7
7	DNA damage_G2 checkpoint in response to DNA mismatches	2.702E-03	CDC25C, Brca1, MSH3
8	SHH signaling in colorectal cancer	2.971E-03	GLI-3R, FOXM1, GLI-3
9	DNA damage_p53 activation by DNA damage	3.751E-03	P53DINP1a, Brca1, PP2A catalytic, AATF (Che-1)
10	HSP22 and BAG-3-dependend macroautophagy in Huntington's disease	8.428E-03	Sequestosome 1(p62), APG12
11	Immune response_Antigen presentation by MHC class I, classical pathway	1.403E-02	NPEPPS, HSP70, Bleomycin hydrolase
12	DNA damage_Inhibition of telomerase activity and cellular senescence	1.479E-02	Brca1, PP2A catalytic
13	Induction of mucin secretion in airway goblet cells by purinergic receptors	1.622E-02	HSP70, PP2A catalytic, SK4/IK1
14	Cell cycle_Cell cycle (generic schema)	1.625E-02	CDC25C, Cyclin B
15	Neurogenesis_NGF/ TrkA MAPK-mediated signaling	1.692E-02	RIN, Sequestosome 1(p62), PP2A catalytic, RIT
16	Apoptosis and survival_Regulation of apoptosis by mitochondrial proteins	1.746E-02	OMA1, APG12, PP2A catalytic, VDAC 1
17	Ubiquitin-proteasome system in Huntington's disease	1.777E-02	HIP-2, PSME3
18	Proteolysis_Putative ubiquitin pathway	1.934E-02	HSP70, SKP1

19	Cytoskeleton remodeling_CDC42 in cellular processes	1.934E-02	COPG2, PARD6
20	Prolactin/ JAK2 signaling in breast cancer	2.097E-02	SK4/IK1, CAML
21	Inhibition of oligodendrocyte precursor cells differentiation by Wnt signaling in multiple sclerosis	2.266E-02	GLI-3R, GLI-3
22	CREB1-dependent transcription deregulation in Huntington's Disease	2.440E-02	CTIP2, GRP75
23	Cell adhesion_Endothelial cell contacts by junctional mechanisms	2.440E-02	Alpha-catenin, Connexin 43
24	DeltaF508-CFTR traffic / Sorting endosome formation in CF	2.619E-02	VPS45A, Vps25
25	Transcription_Negative regulation of HIF1A function	2.681E-02	HSP70, HSPA4, SKP1
26	Development_Positive regulation of STK3/4 (Hippo) pathway and negative regulation of YAP/TAZ function	2.886E-02	PP2A cat (alpha), Alpha-catenin, Alpha-1 catenin
27	Role of IFN-beta in activation of T cell apoptosis in multiple sclerosis	3.188E-02	PP2A cat (alpha), PP2A catalytic
28	Development_Positive regulation of WNT/Beta-catenin signaling in the cytoplasm	3.433E-02	PP2A catalytic, Alpha-1 catenin, USP25
29	DNA damage_Intra S-phase checkpoint	3.433E-02	Brca1, Rad50, GCN5
30	Cell cycle_Start of DNA replication in early S phase	3.591E-02	DRF1, PP2A catalytic
31	Cytoskeleton remodeling_Reverse signaling by Ephrin-B	3.591E-02	Tau (MAPT), FAP-1
32	G-protein signaling_RhoA inhibition	3.666E-02	Cyclin B, Alpha-catenin, PARD6
33	Cell cycle_Spindle assembly and chromosome separation	3.800E-02	Cyclin B, Importin (karyopherin)-beta
34	Microsatellite instability in colorectal cancer	4.013E-02	RFX5, MSH3
35	Development_Negative regulation of WNT/Beta-catenin signaling in the cytoplasm	4.155E-02	KLHL12, PP2A catalytic, Alpha-1 catenin
36	Development_NOTCH signaling activation	4.155E-02	HDAC3, O-fucose, GCN5
37	DNA damage_Double-strand break repair via homologous recombination	4.412E-02	SPIDR, Brca1, PALB2
38	Cell cycle_The metaphase checkpoint	4.453E-02	CENP-H, NSL1

39	G-protein signaling RhoB inhibition	4.453E-02	SNX27, Casein kinase I
40	E-cadherin signaling and its regulation in gastric cancer	4.453E-02	VAV-3, Alpha-catenin
41	Immune response Regulation of T cell function by CTLA-4	4.679E-02	PP2A cat (alpha), PP2A catalytic
42	IL-6 signaling in colorectal cancer	4.679E-02	HSP70, Cyclin B
43	Role of alveolar cell senescence in COPD	4.679E-02	PP2A cat (alpha), PP2A catalytic
44	Cell cycle Regulation of G1/S transition (part 1)	4.910E-02	Brca1, PP2A catalytic
45	Leukotriene 4 biosynthesis and metabolism	5.382E-02	CYP4F3, CYP4F2
46	DNA damage Classical NHEJ mechanism of DSBs repair	5.382E-02	Brca1, XRCC4
47	Stem cells Response to hypoxia in glioblastoma stem cells	5.382E-02	Nucleophosmin, SMAD5
48	Transcription Role of heterochromatin protein 1 (HP1) family in transcriptional silencing	5.382E-02	HP1, HP1 beta
49	Development Hedgehog signaling	5.809E-02	KIF3A, PP2A catalytic, GLI-3
50	Signal transduction WNT/Beta-catenin signaling in tissue homeostasis	5.871E-02	CDC25C, Connexin 43

Table S6. SLC25A16 differentially expressed genes pathway developed by MetaCore. Pro-oncogenic action of androgen receptor was significant correlated in breast cancer disease

#	Maps	pValue	Network Objects from Active Data
1	Pro-oncogenic action of Androgen receptor in breast cancer	2.114E-04	Androgen receptor, HNF3-alpha, PIP, TMF1
2	Development_Positive regulation of WNT/Beta-catenin signaling in the nucleus	2.957E-04	Sirtuin1, Casein kinase I alpha, beta-TrCP, CARF, FOXP1
3	Transport_Clathrin-coated vesicle cycle	3.380E-04	SAR1A, VTI1A, EEA1, Rabaptin-5, SAR1
4	DeltaF508-CFTR traffic / ER-to-Golgi in CF	3.729E-04	Sec24, SAR1A, SAR1
5	wtCFTR traffic / ER-to-Golgi (normal)	3.729E-04	Sec24, SAR1A, SAR1
6	Leucine, isoleucine and valine metabolism.p.2	5.544E-04	HMGCS2, ACDSB, GABT, MMSA, MCCC2
7	Development_Androgen receptor in reproductive system development	5.874E-04	Amphiregulin, Androgen receptor, EEA1, CYP19, SP1
8	Development_Beta-catenin destruction complex signaling	7.116E-04	Casein kinase I alpha, beta-TrCP, Skp2/TrCP/FBXW
9	Putative role of Estrogen receptor and Androgen receptor signaling in progression of lung cancer	1.475E-03	ESR1 (nuclear), Androgen receptor, ESR1 (membrane), CYP19
10	DeltaF508-CFTR traffic / Sorting endosome formation in CF	1.508E-03	EEA1, Rabaptin-5, STAM2
11	O-glycan biosynthesis	2.005E-03	GALNT10, GALNTL2, GALNT7, GALNT4
12	Regulation of lipid metabolism_RXR-dependent regulation of lipid metabolism via PPAR, RAR and VDR	2.055E-03	HMGCS2, CYP19, SP1
13	Development_Extraembryonic differentiation of embryonic stem cells	3.212E-03	GCMA, HNF3-alpha, Transferrin
14	Cell cycle_ESR1 regulation of G1/S transition	3.212E-03	ESR1 (nuclear), Skp2/TrCP/FBXW, SP1
15	WNT signaling in gastric cancer	3.483E-03	Casein kinase I alpha, beta-TrCP, Skp2/TrCP/FBXW
16	PR action in breast cancer: stimulation of cell growth and proliferation	3.483E-03	ESR1 (nuclear), ESR1 (membrane), SP1
17	Immune response_Distinct metabolic pathways in naive and effector CD8+ T cells	3.606E-03	DHE4, DHE3, SLC38A1, Fyn
18	Cell cycle_Regulation of G1/S transition (part 1)	4.064E-03	beta-TrCP, Skp2/TrCP/FBXW, SP1

19	Breast cancer (general schema)	5.042E-03	ESR1 (nuclear), Androgen receptor, ESR1 (membrane)
20	TMPRSS2-ERG fusion in Prostate Cancer	6.963E-03	Androgen receptor, C21orf13, SP1
21	Canonical Leptin pathways in breast cancer	7.393E-03	ESR1 (nuclear), CYP19, SP1
22	Gamma-aminobutyrate (GABA) biosynthesis and metabolism	9.772E-03	DHE4, GABT, DHE3
23	Signal transduction_Angiotensin II/ AGTR1 signaling via p38, ERK and PI3K	1.039E-02	MSK1/2 (RPS6KA5/4), ATP7A, Fyn, SP1
24	Blood coagulation_GPVI-dependent platelet activation	1.083E-02	VAV-3, SNAP-23, Fyn
25	Brca1 and Brca2 in breast cancer	1.115E-02	ESR1 (nuclear), ESR1 (membrane)
26	Ethanol/Acetaldehyde-dependent stimulation of MMP-9 expression in HCC	1.115E-02	beta-TrCP, Skp2/TrCP/FBXW
27	Sphingolipid metabolism	1.349E-02	CERT, SPP lyase, SPT2, SMS1
28	Oxidative stress_Role of Sirtuin1 and PGC1-alpha in activation of antioxidant defense system	1.441E-02	Sirtuin1, PRDX3, SP1
29	EGF- and HGF-dependent stimulation of metastasis in gastric cancer	1.481E-02	VAV-3, SP1
30	Role of Huntingtin in regulation of BDNF in Huntington's disease	1.481E-02	Calcineurin A (catalytic), SP1
31	wtCFTR traffic / Sorting endosome formation (normal)	1.481E-02	EEA1, Rabaptin-5
32	Immune response_Fc epsilon RI pathway: signaling through Fyn and PI3K	1.506E-02	SNAP-23, Fyn, Rab-27B
33	Cytoskeleton remodeling_ESR1 action on cytoskeleton remodeling and cell migration	1.614E-02	ESR1 (nuclear), ESR1 (membrane)
34	Propionate metabolism p.2	1.642E-02	GABT, MMSA, SUCLG2
35	Ovarian cancer (main signaling cascades)	1.784E-02	ESR1 (nuclear), Androgen receptor, SP1
36	Metabolism in pancreatic cancer cells	1.858E-02	DHE4, DHE3, SLC38A1
37	Putative pathways of hormone action in neurofibromatosis type 1	1.892E-02	ESR1 (nuclear), ESR1 (membrane)
38	A proinflammatory phenotype of senescent alveolar epithelial cells in COPD	1.892E-02	Sirtuin1, MSK1
39	Transport_RAB5A regulation pathway	1.892E-02	Rabaptin-5, RUFY2

40	DNA damage_p53 activation by DNA damage	2.010E-02	Sirtuin1, MDM4, P53DINP1a
41	CAR signaling via cross-talk / Human Version	2.039E-02	ESR1 (nuclear), CYP2B6
42	CAR signaling via cross-talk / Rodent version	2.190E-02	ESR1 (nuclear), CYP2B6
43	Development_Positive regulation of STK3/4 (Hippo) pathway and negative regulation of YAP/TAZ function	2.251E-02	MPP5, beta-TrCP, Skp2/TrCP/FBXW
44	Mitogenic action of Estradiol / ESR1 (nuclear) in breast cancer	2.345E-02	ESR1 (nuclear), SP1
45	Cell cycle_Role of SCF complex in cell cycle regulation	2.505E-02	beta-TrCP, Skp2/TrCP/FBXW
46	Histone deacetylases in Prostate Cancer	2.505E-02	Sirtuin1, Androgen receptor
47	Immune response_BAFF-induced non-canonical NF-kB signaling	2.670E-02	beta-TrCP, Skp2/TrCP/FBXW
48	DNA damage_Brca1 as a transcription regulator	2.670E-02	ESR1 (nuclear), SP1
49	Transcription_Ligand-dependent activation of the ESR1/SP pathway	2.670E-02	ESR1 (nuclear), SP1
50	Role of histone modificators in progression of multiple myeloma	2.670E-02	UTX, SP1

Table S7. Gene Pairs of SLC25A16 from STRING Database

No	ProteinID_ENSP00000-	Protein 1	ProteinID_ENSP00000-	Protein 2
1	370989	CD274	335062	PDCD1
2	370989	CD274	476815	SLC25A16
3	342056	CS	362730	MTCH1
4	342056	CS	348299	TRIM13
5	342056	CS	476815	SLC25A16
6	431822	FAU	476815	SLC25A16
7	431822	FAU	447001	RPL18
8	362730	MTCH1	342056	CS
9	362730	MTCH1	476815	SLC25A16
10	303222	MTCH2	476815	SLC25A16
11	417898	PAEP	476815	SLC25A16
12	335062	PDCD1	476815	SLC25A16
13	335062	PDCD1	370989	CD274
14	447001	RPL18	431822	FAU
15	447001	RPL18	476815	SLC25A16
16	476815	SLC25A16	303222	MTCH2
17	476815	SLC25A16	305069	TMEM192
18	476815	SLC25A16	335062	PDCD1
19	476815	SLC25A16	342056	CS
20	476815	SLC25A16	348299	TRIM13
21	476815	SLC25A16	362730	MTCH1
22	476815	SLC25A16	370989	CD274
23	476815	SLC25A16	417898	PAEP
24	476815	SLC25A16	431822	FAU
25	476815	SLC25A16	447001	RPL18
26	305069	TMEM192	476815	SLC25A16
27	348299	TRIM13	342056	CS
28	348299	TRIM13	476815	SLC25A16

Table S8. Gene Pairs of GNPDA1 from STRING Database

No	ProteinID_ENSP00000-	Protein 1	ProteinID_ENSP00000-	Protein 2
1	391596	AMDHD2	216410	GNPNAT1
2	391596	AMDHD2	253778	GFPT2
3	391596	AMDHD2	295448	GNPDA2
4	391596	AMDHD2	349860	GFPT1
5	391596	AMDHD2	405573	GPI
6	391596	AMDHD2	473348	H6PD
7	391596	AMDHD2	423674	GNPDA1
8	455561	AMDHD2-2	216410	GNPNAT1
9	455561	AMDHD2-2	253778	GFPT2
10	455561	AMDHD2-2	295448	GNPDA2
11	455561	AMDHD2-2	349860	GFPT1
12	455561	AMDHD2-2	405573	GPI
13	455561	AMDHD2-2	423674	GNPDA1
14	455561	AMDHD2-2	473348	H6PD
15	455561	AMDHD2-2	494664	HK1
16	349860	GFPT1	216410	GNPNAT1
17	349860	GFPT1	253778	GFPT2
18	349860	GFPT1	295448	GNPDA2
19	349860	GFPT1	318318	MPI
20	349860	GFPT1	473348	H6PD
21	349860	GFPT1	391596	AMDHD2
22	349860	GFPT1	494664	HK1
23	349860	GFPT1	423674	GNPDA1
24	349860	GFPT1	455561	AMDHD2-2
25	349860	GFPT1	405573	GPI
26	253778	GFPT2	216410	GNPNAT1
27	253778	GFPT2	473348	H6PD
28	253778	GFPT2	391596	AMDHD2
29	253778	GFPT2	494664	HK1
30	253778	GFPT2	318318	MPI
31	253778	GFPT2	295448	GNPDA2
32	253778	GFPT2	423674	GNPDA1
33	253778	GFPT2	349860	GFPT1
34	253778	GFPT2	455561	AMDHD2-2
35	253778	GFPT2	405573	GPI
36	423674	GNPDA1	216410	GNPNAT1
37	423674	GNPDA1	253778	GFPT2
38	423674	GNPDA1	295448	GNPDA2
39	423674	GNPDA1	318318	MPI
40	423674	GNPDA1	349860	GFPT1

41	423674	GNPDA1	391596	AMDHD2
42	423674	GNPDA1	405573	GPI
43	423674	GNPDA1	494664	HK1
44	423674	GNPDA1	473348	H6PD
45	423674	GNPDA1	455561	AMDHD2-2
46	295448	GNPDA2	216410	GNPNAT1
47	295448	GNPDA2	253778	GFPT2
48	295448	GNPDA2	473348	H6PD
49	295448	GNPDA2	318318	MPI
50	295448	GNPDA2	494664	HK1
51	295448	GNPDA2	405573	GPI
52	295448	GNPDA2	349860	GFPT1
53	295448	GNPDA2	423674	GNPDA1
54	295448	GNPDA2	391596	AMDHD2
55	295448	GNPDA2	455561	AMDHD2-2
56	216410	GNPNAT1	391596	AMDHD2
57	216410	GNPNAT1	473348	H6PD
58	216410	GNPNAT1	405573	GPI
59	216410	GNPNAT1	455561	AMDHD2-2
60	216410	GNPNAT1	494664	HK1
61	216410	GNPNAT1	295448	GNPDA2
62	216410	GNPNAT1	423674	GNPDA1
63	216410	GNPNAT1	253778	GFPT2
64	216410	GNPNAT1	349860	GFPT1
65	405573	GPI	216410	GNPNAT1
66	405573	GPI	253778	GFPT2
67	405573	GPI	295448	GNPDA2
68	405573	GPI	318318	MPI
69	405573	GPI	349860	GFPT1
70	405573	GPI	391596	AMDHD2
71	405573	GPI	455561	AMDHD2-2
72	405573	GPI	423674	GNPDA1
73	405573	GPI	494664	HK1
74	405573	GPI	473348	H6PD
75	473348	H6PD	216410	GNPNAT1
76	473348	H6PD	253778	GFPT2
77	473348	H6PD	295448	GNPDA2
78	473348	H6PD	318318	MPI
79	473348	H6PD	349860	GFPT1
80	473348	H6PD	391596	AMDHD2
81	473348	H6PD	405573	GPI
82	473348	H6PD	423674	GNPDA1
83	473348	H6PD	455561	AMDHD2-2

84	473348	H6PD	494664	HK1
85	494664	HK1	216410	GNPNAT1
86	494664	HK1	253778	GFPT2
87	494664	HK1	295448	GNPDA2
88	494664	HK1	318318	MPI
89	494664	HK1	349860	GFPT1
90	494664	HK1	405573	GPI
91	494664	HK1	423674	GNPDA1
92	494664	HK1	455561	AMDHD2-2
93	494664	HK1	473348	H6PD
94	318318	MPI	253778	GFPT2
95	318318	MPI	295448	GNPDA2
96	318318	MPI	473348	H6PD
97	318318	MPI	423674	GNPDA1
98	318318	MPI	349860	GFPT1
99	318318	MPI	494664	HK1
100	318318	MPI	405573	GPI
